# Performance of an artificial intelligence system versus endoscopists for interrogation of scars after piecemeal endoscopic mucosal resection

**DOI:** 10.1055/a-2840-7149

**Published:** 2026-05-04

**Authors:** Oswaldo Ortiz, Ricard Prat, Guillem Soy, Alex Bofill, Joan Llach, Maria Daca-Alvarez, Francesc Balaguer, Angelo Brunori, Hardeep Kumari, Romina Vergara, Liseth Rivero-Sánchez, Maria Pellisé

**Affiliations:** 1 Hospital Clinic Barcelona, Gastroenterology Department, Endoscopy Unit 16724 Universitat de BarcelonaBarcelonaSpain; 2 Faculty of Medicine and Health Sciences 16724 Universitat de BarcelonaBarcelonaBarcelonaSpain; 3 Institut d'Investigacions Biomediques August Pi i Sunyer (IDIBAPS)BarcelonaSpain

**Keywords:** Endoscopy Lower GI Tract, Polyps / adenomas / ..., Diagnosis and imaging (inc. chromoendoscopy, NBI, iSCAN, FICE, CLE...), Endoscopic resection (polypectomy, ESD, EMRc, ...)

## Abstract

**Background and study aims:**

Detection and management of recurrence after piecemeal endoscopic mucosal resection (p-EMR) of large colorectal polyps is crucial for preventing post-colonoscopy colorectal cancer. Colonoscopy assisted with artificial intelligence systems for lesion detection (CADe) has shown efficacy in identifying polyps, particularly small lesions (< 10 mm). Our aim was to evaluate sensitivity and negative predictive value of CADe compared with endoscopists in detecting recurrence after p-EMR.

**Methods:**

Sixty-six pseudo anonymized high-quality videos (25 with recurrence and 15 with previously clipped defects) of > 15 mm polyps post-EMR scars were assessed. Recurrence size was < 10 mm in 19 of 25 cases (76.0%). Six endoscopists—three experts and three non-experts—predicted recurrence (yes/no) with confidence level (high/low). The same videos were analyzed with CAD-e detection mode superimposed. The gold standard was histopathology.

**Results:**

Endoscopists had higher sensitivity than CADe (96.0% vs 72.0%; *P* = 0.03). Experts performed better than non-experts in ruling out recurrence (specificity: 92.7% vs 58.5%; *P* = 0.001) and accuracy (92.4% vs 72.7%; *P* < 0.001). In non-clipped defects, endoscopists maintained higher sensitivity than CADe (95.0% vs 65.0%; *P* = 0.03). Concordance was moderate overall (Kappa 0.57) but substantial among experts (0.70) and when excluding clipped defects (0.61)

**Conclusions:**

Endoscopists outperformed CADe in interrogating post-EMR scars. Training CADe with images of post-polypectomy scars may further improve its performance.

## Introduction

Piecemeal endoscopic mucosal resection (p-EMR) remains the cornerstone for the resection of suspected benign non-pedunculated ≥ 20 mm colorectal lesions according to guidelines 1 . The main flaw of p-EMR is its relatively high recurrence rates, even in experts’ hands 2 3 and despite several advances such as thermal ablation of the resection margins 4 .

The remaining recurrent lesions that could evolve into up to 11% of post-colonoscopy colorectal cancers 5 6 are usually diminutive sessile or flat lesions with similar morphological characteristics than small/diminutive polyps and are relatively easy to resect, highlighting the importance of an adequate and exhaustive interrogation of post-EMR scars sites when looking for recurrence 7 . However, evaluation of p-EMR scars may be very challenging in some scenarios, such as presence of clip artifacts, especially when clip artifacts—areas of normal pit pattern distorted by clip placement—mimic recurrence 8 .

Increasing evidence from meta-analyses and randomized controlled trials (RCTs) shows that artificial intelligence-aided (AI) colonoscopy for lesion detection improves colonoscopy performance in average- and high-risk populations 9 10 11 . However, there is no evidence of its role in detecting recurrence in post-polypectomy scars. Therefore, our aim was to compare endoscopist performance versus AI (CAD-EYE) systems evaluation for inspection of scar after resection of non-pedunculated lesions.

## Patients and methods

### Study design and participants

**Fig. 1:**
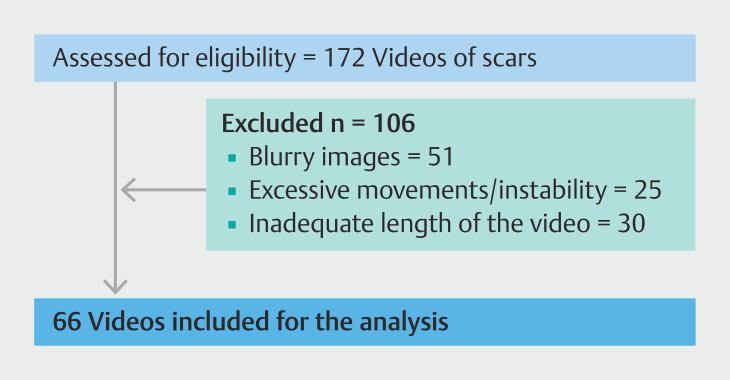
Flowchart for video selection.

An observational post-hoc analysis of a RCT was designed (NCT04899700) 12 . One hundred seventy-two videos of surveillance colonoscopies after p-EMR of ≥15 mm non-pedunculated colorectal lesions evaluated using ELUXEO 7000 (Fujifilm, Tokyo, Japan) with high-definition white light endoscopy (WLE), blue light imaging, and linked color imaging were recorded. After exclusions based on quality and avoiding blurry, too many movements, and ensuring an appropriate length of the video (minimum length of more than 30 seconds), 66 high-definition videos (25 with histopathological recurrence) were finally included for analysis ( Fig. 1 ). The proportion of videos with recurrence was estimated according to prevalence of disease in our cohort (30%). p-EMR scars were inspected with different light modalities and recorded in short videos during a randomized crossover trial 12 (NCT04899700) performed in an academic center. Biopsies were obtained from each scar and histopathology diagnosis was used as the gold standard according to Vienna criteria 13 . Adenomatous histology was defined as any lesion with tubular and/or villous components. Non-adenomatous was defined as sessile serrated, hyperplastic, and/or traditional serrated adenoma histopathology.

Six endoscopists (3 experts and 3 non-experts) blinded to the in situ and histological assessment were asked to make an off-line assessment of the post-EMR defects. Expert endoscopists were defined based on European Society of Gastrointestinal Endoscopy guidelines as those endoscopists with an adenoma detection rate (ADR) in colonoscopy-based screening >25% 14 and/or an ADR in fecal immunochemical test-based screening >45% 15 . Non-expert endoscopists were gastroenterologists without specific expertise in the field but with training in basic upper gastrointestinal and colonoscopy procedures with a personal experience of at least 300 upper and 300 lower gastrointestinal endoscopies according to European quality measures guidelines 16 .

### Procedures

#### Endoscopist assessment

Pseudo-anonymized videos were sent to the observers through a cloud where they were stored and codified. Endoscopists had to predict presence of recurrence (yes or no) with a level of confidence (high or low). Inflammatory changes and/or clip artifact were also collected as normal/inflammatory tissue. Data were collected through an online platform.

Recurrence was counted as positive for endoscopists prediction in all high- and low-confidence cases.

#### Artificial intelligence assessment

CAD-EYE(ELUXEO 7000; Fujifilm, Tokyo, Japan), a commercial AI system based on deep learning and trained for colon polyp detection and characterization, was superimposed on the videos to analyze its capability to detect recurrences on p-EMR scars. When a suspected lesion was detected, a blue box encircled the area. For the study, an adequate inspection was ensured and defined as follows: inspection with centered scar and adequate stability without bubbles, undigested local debris or local bleeding.

Assessment of computer-aided diagnostic (CADe) detection performance was evaluated by an independent observer. Recurrence was counted as positive when the detection box was activated and fixed on the same lesion for two or more seconds 17 . False positives were those where the detection box was fixed for more than 2 seconds but no adenomatous or serrated histology was found in the histopathology evaluation 17 . The 2-second cut-off was used with the goal of balancing sensitivity with clinical relevance and minimizing overcounting of fleeting, non-actionable detections.

### Outcomes

The primary outcome was to compare overall performance in terms of sensitivity and negative predictive values (NPVs) of endoscopists versus AI (CADe) for evaluation of recurrence in post-polypectomy scars. Secondary outcomes were performance for CADe and endoscopists between different endoscopist categories (experts and non-experts), after exclusion of clipped post-EMR scars at index colonoscopy, between histopathology subcategories (adenoma/non-adenomatous), grade of concordance between observers for detection of recurrence on post-EMR scars, and false positives of CADe in diagnosis of recurrence.

### Statistical analysis

Categorical variables were expressed as frequencies and percentages. Continuous variables as medians and interquartile range (IQR) for non-normal distribution tested with Kolmogorv-Smirnov test. Diagnostic measures such as sensitivity, specificity, positive predictive value (PPV), negative predictive value (NPV), and accuracy were calculated using a 2×2 table. The comparison between CADe versus overall endoscopists and between endoscopist groups (experts versus non-experts) was based on Mc Nemar test considering the paired observations of the analysis. The *P* value represents the comparison of these observations between both groups. Two-sided *P* <0.05 was considered statistically significant. All measures were expressed as percentages along with 95% confidence intervals (CIs) based on the Wilson method without continuity correction. Overall diagnosis performance of the endoscopist was calculated as follows: The dichotomous outcome (recurrence/no recurrence) for diagnosis of recurrence among the endoscopists was averaged. If the average result defined as the arithmetic mean was >0.5, it was classified as recurrence, and if <0.5, as no recurrence. For comparison between endoscopists, group-level means were calculated: one mean based on observations from expert endoscopists and another mean based on observations from non-experts. Subsequent analyses were conducted using these aggregated group-level results rather than endoscopist individual-level outcomes.

For observer concordance, pairwise agreement with Fleiss Kappa was used to rate concordance of more than two categorical variables. Agreement was measured according to Landis and Koch scale. SPSS statistics for windows software (version 20.0; IBM Corporation, Somers, New York, United States) were used for statistical analysis.

## Results

Sixty-six high-quality videos of post-EMR scars were included and evaluated in the study. Baseline characteristics of scars are shown in Table 1 . There were 25 of 66 recurrences (37.9%). Baseline histology was adenoma in 44 of 66 (66.7%) and non-adenomatous in 22 of 66 (33.3%). Clips artifacts confirmed by histopathology were seen in eight of 66 (12.1%) and clip placement on baseline procedure was seen in 15 of 66 (22.7%).

**Table:** **Table 1** Baseline characteristics of post-EMR scars.

Characteristics	
Scar size median (IQR 25%–75%)	12.0 (10.0–15.0)
Location n (%)	Cecum 5 (7.6%)
	Ascending colon 19 (28.8%)
	Hepatic flexure 8 (12.1)
	Transverse colon 16 (24.2%)
	Descending colon 4 (6.1%)
	Sigmoid 3 (4.5%)
	Rectum 11 (16.7%)
Time from initial EMR to surveillance (months) median (IQR 25–75%)	10.5 (6.9–17.5)
Thermal ablation margins at index procedure n (%)	17/66 (25.76%)
Presence of recurrence n (%)	25/66 (37.9%)
Morphology of recurrence n (%)	
Sessile (Is)	15/25 (60.0%)
Flat-elevated (IIa)	10/25 (40.0%)
Recurrence size n (%)	
<5 mm	10/25 (40.0%)
5–9 mm	9/25 (36.0%)
>10 mm	6/25 (24.0%)
Baseline histology n (%)	
Adenoma	44/66 (66.7%)
Non-adenomatous	22/66 (33.3%)
Clips placement on baseline procedure n (%)	15/66 (22.7%)
Cip artifacts confirmed by histopathology n(%)	8/66 (12.1%)
EMR, endoscopic mucosal resection; IQR, interquartile range.	

### CADe performance compared with endoscopists in detecting recurrence after p-EMR.

**Fig. 2:**
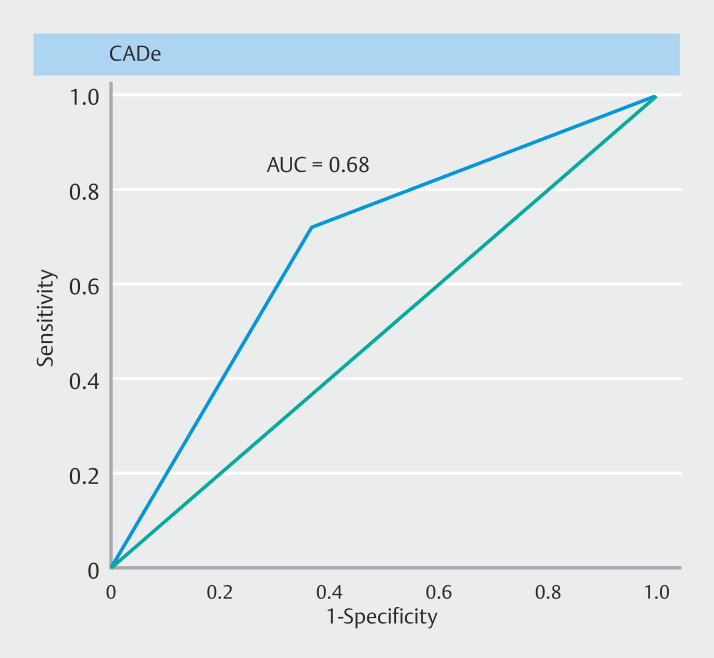
Area under ROC for CADe performance.

**Fig. 3:**
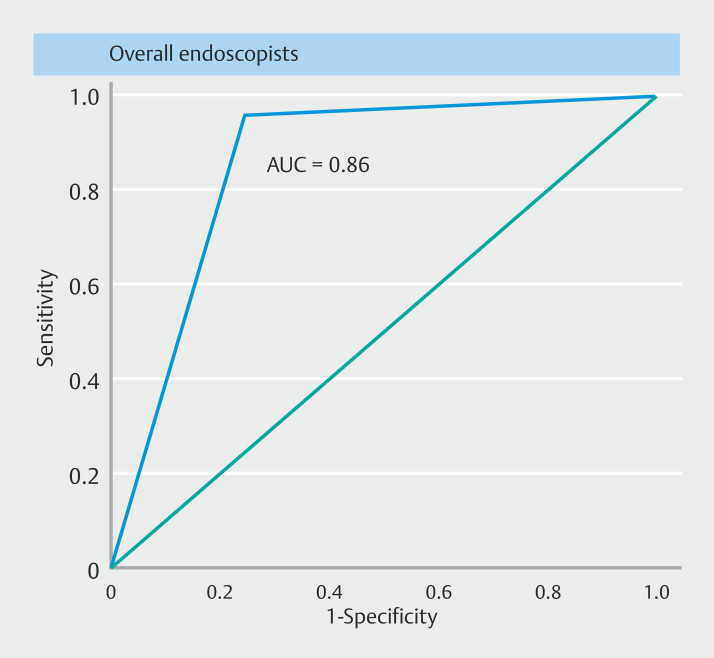
Area under ROC curve for overall endoscopist performance.

Performance of endoscopists compared with CADe is shown in Table 2 . Endoscopists demonstrated significantly higher sensitivity for detecting recurrence: 24 of 25 (96.0%) (95% CI 80.5–99.3) vs 18 of 25 (72.0%) (95%CI 52.4–85.7); *P* = 0.03 and higher NPV: 31 of 32 (96.9%) (95%CI 84.3–99.5) vs 26 of 33 (78.8%) (95% CI 62.3–89.3). No significant differences in terms of specificity between endoscopists (31 of 41; 75.6%) (95% CI 60.6–86.2) and CADe (26 of 41; 63.4%) (95% CI 48.1–76.4) ( *P* = 0.23) were found. False-positive rates for CADe versus endoscopists were 15 of 41 (36.6%) and 10 of 41 (24.4%), respectively. The area under receiver operating characteristic curve for CADe and endoscopists are shown in Fig. 2 and Fig. 3 .

**Table:** **Table 2** Performance diagnostic measures of endoscopists compared with CADe.

	CADe	Endoscopists	*P* value
Sensitivity	** 18/25 (72.0%) [52.4 ** – **85.7]**	** 24/25 (96.0%) [80.5 ** – **99.3]**	**0.03**
Specificity	26/41 (63.4%) [48.1–76.4]	31/41 (75.6%) [60.6–86.2]	0.23
Positive predictive value	18/33 (54.6%) [38.0–70.2]	24/34 (70.6%) [53.8–83.2]	
Negative predictive value	26/33 (78.8%) [62.3–89.3]	31/32 (96.9%) [84.3–99.5]	
Accuracy	44/66 (66.7%) [54.7–76.8]	55/66 (83.3%) [72.6–90.4]	1.00
CADe, computer-aided detection.			

Subanalysis of non-expert endoscopists still showed high sensitivity compared with CADe 24 of 25 (96.0%) (95%CI 80.5–99.3) vs 18 of 25 (72.0%) (95%CI 80.6–97.5); *P* = 0.03, which led to a non-significant greater accuracy for non-experts 48 of 66 (72.7%) (95% CI 61.0–82.0) compared with CADe 44 of 66 (66.7%) (95% CI 54.7–76.8) *P* = 0.08 ( Table 3 ).

**Table:** **Table 3** Performance diagnostic measures between CADe and non-expert endoscopists.

	CADe	Non-experts endoscopists	*P* value
Sensitivity	** 18/25 (72.0%) [52.4 ** – **85.7]**	** 24/25 (96.0%) [80.5 ** – **99.3]**	0.03
Specificity	26/41 (63.4%) [48.1–76.4]	24/41 (58.5%) [43.4–72.2]	0.75
Positive predictive value	18/33 (54.6%) [38.0–70.2]	24/41 (58.5%) [43.4–72.2]	
Negative predictive value	26/33 (78.8%) [62.3–89.3]	24/25 (96.0%) [80.5–99.3]	
Accuracy	44/66 (66.7%) [54.7–76.8]	48/66 (72.7%) [61.0–82.0]	0.08
CADe, computer-aided detection.			

Performance measures for each endoscopist for evaluation of post-EMR scars are shown in Supplementary Table 1.

### Secondary objectives

Expert endoscopists demonstrated better performance for ruling out recurrence when compared with non-experts with a significant higher specificity: 38 of 41 (92.7%) (95%CI 80.6–97.5) vs 24 of 41 (58.5%) (95%CI 43.4–72.2; *P* = 0.001) and PPV 23 of 26 (88.5%) (95%CI 71.0–96.0) vs 24 of 41 (58.5%) (95%CI 43.4–72.2), which led to a significant higher accuracy (61 of 66; 92.4%) (95% CI 83.5–96.7) vs 48 of 66 (72.7%) (95% CI 61.0–82.0); *P* <0.0001( Table 4 ).

**Table:** **Table 4** Performance diagnostic measures of experts endoscopists compared with non-experts.

	Experts	Non-experts	*P* value
Sensitivity	23/25 (92.0%) [75.0–97.8]	24/25 (96.0%) [80.5–99.3]	1.00
Specificity	** 38/41 (92.7%) [80.6–97.5] **	** 24/41 (58.5%) [43.4–72.2] **	**0.001**
Positive predictive value	** 23/26 (88.5%) [71.0–96.0] **	** 24/41 (58.5%) [43.4–72.2] **	
Negative predictive value	38/40 (95.0%) [83.5–98.6]	24/25 (96.0%) [80.5–99.3]	
Accuracy	** 61/66 (92.4%) [83.5 ** – **96.7]**	** 48/66 (72.7%) [61.0 ** – **82.0]**	**0.0001**

After excluding previously clipped post-EMR defects, endoscopists maintained higher sensitivity for detection of recurrence compared with CADe (95.0%) (95%CI 76.4–99.1) vs 65.0% (95% CI 43.3–81.8) *P* = 0.03 (Supplementary Table 2).

### Histopathology categories

No significant differences in performance measures when comparing CADe and endoscopists were obtained according to histopathology categories: adenoma and non-adenomatous lesions (Supplementary Table 3 and Supplementary Table 4).

### High- vs low-confidence cases

In 277 of 396 cases (70.0%), a high-confidence optical diagnosis was made with an overall sensitivity of 94.2% and NPV of 94.0%. Subanalysis based on endoscopist expertise showed that expert endoscopists were more prone to high-confidence diagnosis compared with non-experts (167 vs 110 cases) and better accuracy (91.6% vs 78.2%). A summary of diagnostic performance in high- and low-confidence cases between endoscopist categories and individual performance of endoscopists is shown in Table 5 and Supplementary Table 5 .

**Table:** **Table 5** Performance metrics between CADe, endoscopists overall, and high and low confidence cases.

	CADe	Endoscopists overall	Endoscopists high confidence	Endoscopists low confidence
Sensitivity	18/25 (72.0%)	24/25 (96.0%)	114/121 (94.2%)	24/29 (82.8%)
Specificity	26/41 (63.4%)	31/41 (75.6%)	125/156 (80.1%)	56/88 (63.6%)
Positive predictive value	18/33 (54.6%)	24/34 (70.6%)	114/145 (78.6%)	24/56 (42.9%)
Negative predictive value	26/33 (78.8%)	31/32 (96.9%)	125/133 (94.0%)	56/74 (75.7%)
Accuracy	44/66 (66.7%)	55/66 (83.3%)	239/277 (86.3%)	80/119 (67.2%)
CADe, computer-aided detection.				

### Concordance and agreements

Moderate agreement was obtained for all six observers with an overall Kappa of 0.57 (95% CI 0.489–0.614; *P* <0.0001). A slight increase in agreement was observed when excluding previously clipped defects Kappa 0.61; (95% CI 0.54–0.68). Subanalysis according to endoscopist experience showed a substantial agreement between experts Kappa: 0.70 (95% CI 0.56–0.85; *P* <0.0001) and still moderate agreement for non-experts Kappa: 0.52 (95% CI 0.38–0.66; *P* <0.0001).

## Discussion

To our knowledge, this observational post-hoc analysis is the first study aimed at exploring potential benefits of CADe in evaluation of post-EMR scars. CADe demonstrated acceptable performance in terms of sensitivity and NPV for evaluation of post-EMR scars, although its diagnostic capabilities appeared to be inferior to endoscopists, particularly dedicated endoscopists. Statistically significant differences in sensitivity between CADe and endoscopists (72.0% vs. 96.0%, *P* = 0.03) suggest that the system may miss a substantial number of recurrent lesions. This limitation is especially relevant, given that three of four recurrences were smaller than 10 mm and 40% of them had flat morphology, requiring high sensitivity for detection of subtle lesions. Also, CADe showed low PPV (54.6%), which indicates a tendency to generate false positives, leading to a potential increase in cognitive load on clinicians and unnecessary interventions.

Remarkably, high sensitivity (>90%) was obtained for all endoscopist evaluations. These results concur with a previous observational study involving 230 scars from an academic center 18 and a recent RCT aimed at comparing WLE to narrow-band imaging (NBI), showing a NBI performance of 96% 19 . High (>90%) NPVs from endoscopists were obtained in our study in line with previous studies 18 19 at achieving the Preservation and Incorporation of Valuable endoscopic Innovations (PIVI) criteria 20 , reinforcing that in case of a bland flat scar with no recurrence by optical diagnosis, no biopsies are needed to confirm the results 1 .

Subanalysis based on endoscopist expertise showed that in comparison, CADe had lower sensitivity compared with non-expert endoscopists, but its specificity was slightly higher, indicating potential utility in reducing false positives among less experienced practitioners. In fact, the comparison between endoscopists based on their expertise showed that expert endoscopists were better than non-experts at ruling out recurrence, with significantly higher specificity, without a significant decrease in sensitivity, leading to a statistically significant improvement in accuracy. The increase in specificity reflects a tendency among non-experts to be cautious and interpret all minor findings as recurrence and highlights the importance of endoscopists training in optical diagnosis of recurrence to reduce the false-positive rate and overtreatment of post-EMR defects.

Importantly, to our knowledge, currently there are no dedicated guidelines or validated training curricula specifically focused on optical diagnosis of residual or recurrent adenoma in post-EMR scars. However, evidence supports that a systematic approach to imaging can achieve high diagnostic accuracy. In particular, Desomer et al. 21 demonstrated that a standardized imaging protocol using high-definition white light (HD-WL) followed by NBI provides excellent diagnostic performance for detecting recurrence at first surveillance after EMR, with a sensitivity of 93.3%, specificity of 94.1%, and NPV of 98.6%. Notably, sensitivity was substantially higher when combining HD-WL with NBI compared with HD-WL alone (93.3% vs 66.7%), and flat recurrence morphology was better detected with NBI. Training in scar assessment should include structured teaching and hands-on instruction in a standardized imaging protocol (systematic scar inspection with HD-WL and NBI) and recognition of subtle, flat recurrence patterns. In addition, given the close endoscopic resemblance between diminutive recurrence and diminutive colorectal polyps, and in the absence of scar-specific curricula, we recommend aligning training with the European Society of Gastrointestinal Endoscopy optical diagnosis curriculum for diminutive colorectal lesion 22 . This curriculum includes participation in validated training courses led by experts, training in established classification systems (Narrow Band Imaging International Colorectal Endoscopic [NICE], Japan NBI Expert Team [JNET], Workgroup serrAted polypS and Polyposis (WASP), and Sano), and competence maintenance through continuous auditing and self-learning, including prospective assessment of at least 60 diminutive colorectal lesions per year and performance monitoring according to PIVI thresholds.

**Fig. 4:**
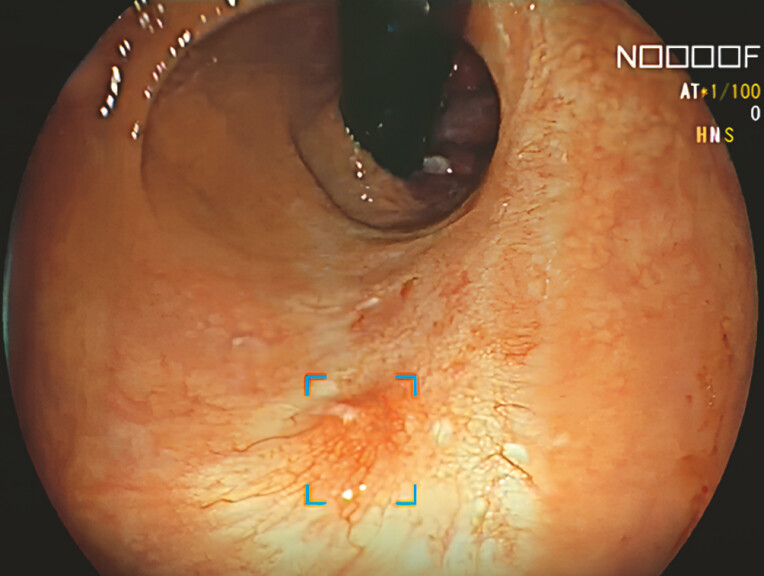
CADe false positive (blue box) over a sessile area on the edge of a scar after piecemeal resection of a rectal lesion. The highlighted area was resected by the endoscopist and the final histopathology was consistent with clip artifact.

Prophylactic placement of clips for closure of the mucosal defect is recommended in certain high-risk cases, considering risk factors for delayed bleeding such as lesion size, proximal location, patient comorbidities, and use of anticoagulants or antiplatelet therapy 23 24 25 . Clip artifacts after prophylactic clip placement on post-EMR scars could affect diagnostic performance of endoscopists ( Fig. 4 ). In the 15 clip artifact cases, the false-positive rate was numerically higher than with CADe compared with endoscopists (40% vs. 20%), underscoring challenges in evaluating scars with clip artifacts. In our study, analyzing only non-clipped scars, we obtained an increase in specificity of 80.7% for all endoscopists (90.3% for experts and 64.5% for non-experts) with an accuracy of 86.3%.

According to histopathology, the system performed particularly poorly in detecting non-adenomatous lesions, with a sensitivity of just 50.0%, possibly due to algorithmic constraints or the less prominent visual features of these lesions. This underscores the challenges of evaluating serrated recurrence in post-EMR scars. These difficulties may be explained by the similarity in appearance between normal mucosa and hyperplastic or serrated lesions, particularly when assessed using WLE. It is important to emphasize that adequate training of CADe systems with hyperplastic and serrated lesions must be addressed and validated to ensure their effectiveness in detecting this type of lesion. However, it is important to note that these results should be taken cautiously, due to the low sample size when comparing histopathology categories.

In high-confidence cases, diagnostic performance of endoscopists in terms of sensitivity and NPV was above 90%, even in the non-expert setting. These findings support the strategy of avoiding random biopsies from post-EMR scars when a high-confidence negative diagnosis of recurrence is made. However, in low-confidence diagnoses, the absolute numbers are too small to draw specific conclusions (particularly in the expert group), although diagnostic performance appears to be lower than in high-confidence cases, supporting a biopsy or resection strategy in these situations.

Interobserver agreement was moderate but improved substantially when considering only expert endoscopists. These findings, in addition to the previously commented higher specificity and accuracy between experts when compared with non-experts, underscore the importance of appropriate training of endoscopists in evaluation of post-EMR scars.

This study has several important methodological strengths. First, it included multiple endoscopists with varying levels of expertise, enhancing external validity and generalizability of the findings across different clinical settings. Second, the direct comparison between CADe and human performance allowed for a robust assessment of diagnostic accuracy under real-world conditions. Notably, this comparison was performed in clinically challenging scenarios, such as post-EMR scars with clip-related artifacts, which are often underrepresented in validation studies but highly relevant to routine practice. Together, these aspects strengthen reliability of the results and support their applicability to everyday endoscopic surveillance. However, it is important to note that our study has several potential drawbacks **.** First, the version of the evaluated CADe system (CAD-EYE from Fujifilm) is not adequately trained with post-EMR scars images. This fact could explain the lack of benefit shown in our results. CADe systems could play a role as a black box, making it difficult to infer decisions, and in some specific settings, for example, when evaluating post-EMR scars, their performance could differ from what is expected. This is important because recommendations on use of CADe systems are gaining importance and it underscores the need to understand the setting in which each CADe system is trained to assess its potential applications and limitations. We believe that training the actual available and commercialized CADe systems with images of polyp recurrences is the most pragmatic way for these systems to become cost-effective and have clinical utility. In routine clinical practice, endoscopists mainly use CADe systems for polyp detection, often without knowing whether a recent polypectomy has been performed in that segment of the colon—especially in the context of multiple resected polyps and several procedures. This, combined with the fact that scars from polyps < 20 mm are very subtle, and recurrences are usually diminutive and closely resemble small polyps, would help endoscopists increase their detection rate, not only of naïve lesions but also of recurrences. However, it must be considered that this training could negatively impact diagnostic performance in polyp detection and potentially increase the number of false positives.

Second, our study is a single-center study performed in an academic setting, leading to a potential decrease in external validation of results, although we included observations from three non-expert gastroenterologists to minimize this bias. Third, the observational nature of the study and the fact that only off-line assessments were performed could lead to a decrease in diagnostic performance that could affect both endoscopists and CADe. We tried to minimize this bias by selecting only videos with a higher standard of quality in terms of length, definition, and centered image. Finally, microscopic recurrence defined as recurrence detected by biopsies but undetectable with the actual optical diagnosis techniques is an issue that needs to be determined in further studies.

## Conclusions

In conclusion, our observational post-hoc analysis failed to demonstrate superiority of a CADe system compared with actual standard evaluation of post-polypectomy scars in terms of sensitivity and negative predictive value using high-definition WLE and virtual chromoendoscopy. CADe may play a complementary role in clinical practice, particularly as a second reader or support tool for less experienced endoscopists and/or on specific scenarios. However, its use as a standalone diagnostic solution is not yet justified. Further algorithmic training and refinement and prospective studies are needed to assess its impact on clinical decision-making and patient outcomes. Finally, proper training of endoscopists remains the cornerstone for evaluation of scars after resection of large polyps.

## Supplementary Material

Supplementary Material Zusatzmaterial
