# Supplementary material for: Performance of an artificial intelligence system versus endoscopists for interrogation of scars after piecemeal endoscopic mucosal resection
Source: Endosc Int Open. 2026 May 4;14:a28407149. doi: 10.1055/a-2840-7149 (PMC13289963; doi:10.1055/a-2840-7149)
Supplement: Supplementary file 1 — Supplementary Material [file 10-1055-a-2840-7149_28542113.pdf]

Supplementary Table 1 Diagnostic performance measures of endoscopists.

	Experts			Non-experts		
	**Endoscopist 1**	**Endoscopist 2**	**Endoscopist 3**	**Endoscopist 4**	**Endoscopist 5**	**Endoscopist 6**
Sensitivity	24/25 (96.0%)	24/25 (96.0%)	22/25 (88.0%)	21/25 (84.0%)	25/25 (100%)	21/25 (84.0%)
Specificity	20/41 (48.8%)	26/41 (63.4%)	29/41 (70.7%)	38/41 (92.7%)	35/41 (85.4%)	35/41 (85.4%)
Positive predictive value	24/45 (53.3%)	24/39 (61.5%)	22/34 (64.7%)	21/24 (87.5%)	25/31 (80.7%)	21/27 (77.8%)
Negative predictive value	20/21 (95.2%)	26/27 (96.3%)	29/32 (90.6%)	38/42 (92.7%)	35/35 (100%)	35/39 (89.7%)
Accuracy	44/66 (66.7%)	50/66 (75.8%)	51/66 (77.3%)	59/66 (89.4%)	60/66 (90.9%)	56/66 (84.9%)

Supplementary Table 2 Diagnostic performance measures between CADe and endoscopists for non-previously clipped scars.

	CADe	Endoscopists	*P* value
Sensitivity	13/20 (65.0%) [43.3-81.8]	19/20 (95.0%) [76.4-99.1]	**0.03**
Specificity	24/31 (77.4%) [60.2-88.6]	25/31 (80.7%) [63.7-90.8]	1.00
Positive predictive value	13/20 (65.0%) [43.3-81.8]	19/25 (76.0%) [56.6-88.5]	
Negative predictive value	24/31 (77.4%) [60.2-88.6]	25/26 (96.2%) [81.1-99.3]	
Accuracy	37/51 (72.6%) [59.1-82.9]	44/51 (86.3%) [74.3-93.2]	0.27

CADe, computer-aided diagnosis.

Supplementary Table 3 Performance measures between CADe and endoscopists for adenomatous lesions.

	CADe	Endoscopists	*P* value
Sensitivity	13/15 (86.7%) [62.1-96.3]	15/15 (100.0%) [79.6-100.0]	1.00
Specificity	17/29 (58.6%) [40.7-74.5]	22/29 (75.9%) [57.9-87.8]	0.18
Positive predictive value	13/25 (52.0%) [33.5-70.0]	15/22 (68.2%) [47.3-83.6]	
Negative predictive value	17/19 (89.4%) [68.6-97.1]	22/22 (100%) [85.1-100.0]	
Accuracy	30/44 (68.2%) [53.4-80.0]	37/44(84.1%) [70.6-92.1]	0.55

CADe, computer-aided diagnosis.

Supplementary Table 5 Diagnostic performance in high/low confidence cases between experts and non-experts endoscopists.

	Non-experts			Experts	
High confidence	Sensitivity	50/52	(96.2%)	64/69	(92.8%)
	Specificity	36/58	(62.1%)	89/98	(90.8%)
	PPV	50/72	(69.4%)	64/73	(87.7%)
	NPV	36/39	(92.3%)	89/94	(94.7%)
	Accuracy	86/110	(78.2%)	153/167	(91.6%)
Low confidence	Sensitivity	20/23	(87.0%)	4/6	(66.7%)
	Specificity	39/65	(60.0%)	17/23	(73.9%)
	PPV	20/46	(43.5%)	4/10	(40.0%)
	NPV	39/53	(73.6%)	17/21	(81.0%)
	Accuracy	59/88	(67.0%)	21/31	(67.7%)

Supplementary Table 4 Performance measures between CADe and endoscopists for non-adenomatous lesions.

	CADe	Endoscopists	*P* value
Sensitivity	5/10 (50.0%) [23.7-76.3]	9/10 (90.0%) [59.6-98.2]	0.13
Specificity	9/12 (75.0%) [46.8-91.1]	9/12 (75.0%) [46.8-91.1]	1.00
Positive predictive value	5/8 (62.5%) [30.6-86.3]	9/12 (75.0%) [46.8-91.1]	
Negative predictive value	9/13 (69.2%) [42.4-87.3]	9/10 (75.0%) [59.6-98.2]	
Accuracy	14/22(63.6%) [43.0-80.3]	18/22(81.8%) [61.5-92.7]	0.21

CADe, computer-aided detection.

NPV, negative predictive value; PPV, positive prediction value.
